# Worse Disease Prognosis Is Associated to an Increase of Platelet-Derived Extracellular Vesicles in Hospitalized SARS-CoV-2 Patients

**DOI:** 10.1155/2022/8074655

**Published:** 2022-07-07

**Authors:** Davide Raineri, Chiara Venegoni, Maria Grazia Calella, Rosanna Vaschetto, Lorenza Scotti, Elena Canciani, Marcello Manfredi, Francesco Gavelli, Luigi Castello, Annalisa Chiocchetti, Giuseppe Cappellano

**Affiliations:** ^1^Department of Health Sciences, Interdisciplinary Research Center of Autoimmune Diseases-IRCAD, Università del Piemonte Orientale, Novara, Italy; ^2^Center for Translational Research on Autoimmune and Allergic Disease-CAAD, Università del Piemonte Orientale, Novara, Italy; ^3^Department of Translational Medicine, Università del Piemonte Orientale, Novara, Italy; ^4^Emergency Department, “Maggiore della Carità” University Hospital, Novara, Italy; ^5^Internal Medicine, AO “Santi Antonio e Biagio e Cesare Arrigo”, Alessandria, Italy

## Abstract

Platelet-derived extracellular vesicles (PLT-EVs), the most abundant circulating EVs, have been found to be increased in several human diseases, including viral infections. Recently, we documented that PLT-EV counts are higher in SARS-CoV-2+ patients, enrolled during the first two waves of COVID-19, occurred in Italy last year, and we suggested PLT-EVs as a biomarker of SARS-CoV-2 infection. The present study is aimed at testing the ability of PLT-EV levels, measured at hospital admission and within one week of hospitalization, to predict patient's outcome. We applied an easy, fast, and reliable method, based on flow cytometry, for the detection of PLT-EVs in unmanipulated blood samples. In a cohort of SARS-CoV-2 patients, enrolled during the third wave of COVID-19 in Italy, we confirmed that PLT-EV counts are higher in comparison to healthy controls. Moreover, their number is not affected by prehospitalization treatment neither with heparin nor with steroids that are recommended by WHO guidelines. Noteworthy, we identified two pattern of patients, those who increased their PTL-EV level during first week and those reducing it. The former group representented more compromised patients, with higher 4C score, and unfavorable outcome. In conclusion, our new findings would suggest that a worse evolution of the disease is linked with increasing PLT-EV levels in the week after hospital admission.

## 1. Introduction

Extracellular vesicles (EVs) are microparticles that bud from all cells' surface and are transported in body fluids. EVs have been classified by size, biogenesis, and cell type of origin; currently, EVs are categorized into three main types: (1) microvesicles (MVs) (100–1000 nm in diameter), (2) exosomes (20–150 nm), and (3) apoptotic blebs (1000–5000 nm) [1, 2]. EVs circulate and act in the extracellular environment and can resist to the enzymatic digestion due to the presence of their lipid membrane, which is highly enriched in cholesterol, sphingomyelin, annexin, phosphatidylserine, and glycosphingolipids [3]. EVs are deeply involved in the mechanisms of cell-to-cell communication, which is based on different processes including horizontal transfer of several molecules [4].

EVs are stable and can be detected in several body fluids such as blood, saliva, urine, and breast milk [5]. By originating from the parental cells, they may resemble the current state of disease because they carry the same molecules (e.g., miRNA, mRNA, and lipid). Therefore, EVs have attracted attention as they represent an easily obtainable object of study, through liquid biopsies. Considering the minimally invasive nature of the sampling process and their easy accessibility, EVs are emerging as diagnostic/prognostic biomarker for several human diseases [6].

According to the International Society for Extracellular Vesicles (ISEV) guidelines, several methods have been recommended to identify, characterize, and isolate EVs [7]. These include ultracentrifugation, size-exclusion chromatography, immunoaffinity capture, and microfluidics. These methods involve different steps of centrifugation, precipitation, and ultracentrifugation, and they require manipulation of the samples (from blood to plasma) which would represent a stress condition for the cells which in turn might increase EVs release per se; thus, the amount of EVs present in a sample may be distorted. In our recent publication, we established a quick method (1 hr) for the quantification of platelet- (PLT-) derived EVs in fresh blood without sample's manipulation using flow cytometry [8].

At the beginning of 2020, coronavirus disease-19 (COVID-19) was declared a pandemic by the World Health Organization (WHO). Nowadays, it is well recognized as a complex disease involving high levels of inflammation and thrombosis, and PLT hyperactivation correlates with disease severity [9]. Though many efforts have been done, few biomarkers for COVID-19 have been identified; however, these are individually poorly specific, and novel biomarkers are needed to better predict patient outcome.

We recently showed that PLT-EV counts were increased in two independent cohorts of SARS-CoV-2 patients hospitalized during the first two waves of COVID-19 pandemic, occurring in Italy between April 2020 and December 2020. Therefore, we suggested that PLT-EVs could be used as a biomarker of SARS-CoV-2 infection [8]. Given that we applied a fast and reliable method for EV count and easy to implement in hospital clinical laboratories, in this study, we aimed at testing the predictive value of PLT-EVs in COVID-19 evolution during the first week of COVID-19 patient hospitalization.

## 2. Methods

### 2.1. Patients

During the third wave of COVID-19 pandemic occurred between December 2020 and April 2021, we enrolled all SARS-CoV-2+ patients hospitalized at University Hospital “Maggiore della Carità” (Novara, Italy) with blood samples available and who signed the informed consent to participate in the study. SARS-CoV-2 infection was confirmed by reverse-transcriptase polymerase chain reaction (RT-PCR). Blood was withdrawn into sodium citrate collection tubes at two different time points: at the time of admission at the emergency room (T0) and after one week of hospitalization (T7). Blood parameters were measured by using the Sysmex XN-2000™ Hematology System (Sysmex, Kobe, Japan) at Hospital “Maggiore della Carità” at the time of enrollment, while EV analysis was performed within few hours after blood withdrawal at the indicated time points above. Moreover, information on gender, age, comorbidities, and prehospitalization treatment with steroids and heparin was collected. The 4C score was also calculated to evaluate the mortality risk at T0 of the enrolled patients.

A sample of healthy controls (HC), identified among hospital workers, was also included in the study.

The study was approved by local ethic committee (CE67/20); written informed consent was obtained from the patients or their legal representative.

### 2.2. Flow Cytometry

EVs were quantified by flow cytometry from the whole blood of SARS-CoV-2+ patients, as previously described by us [8]. Briefly, we used a custom kit (Becton and Dickinson, NJ, USA) containing a cationic probe which stains lipophilic membrane of EVs and a viability dye (i.e., phalloidin) which identifies intact and viable circulating EVs. Intact EVs were then stained with a combination of three monoclonal antibodies (Becton and Dickinson, NJ, USA) to detect EVs released by the most abundant cell populations in the blood: leukocyte-derived EVs (CD45+), endothelial-derived EVs (CD31+), and PLT-derived EVs (CD31+ CD41a+). Samples were acquired using FACSymphony A5 (Becton and Dickinson, NJ, USA), and flow cytometry data were analyzed using the FACSDiva software (Becton and Dickinson, NJ, USA). The gating strategy is shown in [8]. The count of EVs/*μ*L was obtained using the following formula:

(1) $$\begin{array}{c} \text{EVs}/\mu \text{L}=\frac{\text{No}.\text{of EV events for a given population}*\text{dilution factor}}{\text{Acquired volume}}. \end{array}$$

### 2.3. Statistical Analysis

Descriptive statistics were used to summarize patients' characteristics. Categorical variables were reported as absolute frequencies and percentages while numerical variables as median and first (Q1) and third quartiles (Q3) since not normally distributes. D'Agostino and Pearson test was used to assess the normality distribution of numerical variables. Mann–Whitney test was used to compare the average platelets' EV count between patients with SARS-CoV-2 and HC and 4C score between subjects increasing or decreasing platelets' EVs between T0 and T7, while Wilcoxon sum rank test was used to compare average platelets' EV count between T0 and T7. Finally, Kruskal-Wallis test was used to compare average platelets' EV count in patients assuming corticosteroid and heparin alone or in combination. All tests performed were two tailed, and the type one error was set to 0.05. All analyses were performed using Prism version 8.4.3.

## 3. Results

We enrolled 78 SARS-CoV-2+ patients hospitalized at University Hospital “Maggiore della Carità” (Novara, Italy).  Table 1 summarizes the demographic and clinical data of the cohort of hospitalized SARS-CoV-2+ patients. PLT-EVs were quantified in unmanipulated blood, as previously described by us [8].

**Figure 1:**
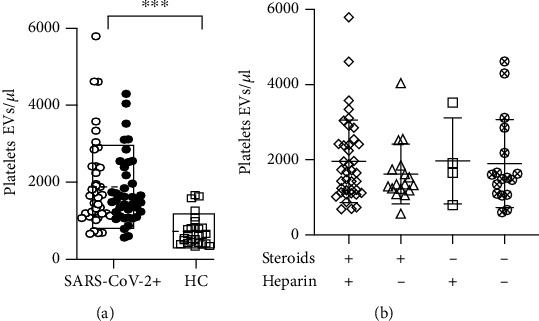
PLT-EV counts are higher in SARS-CoV-2+ patients regardless of heparin and steroid treatments. (a) Dot plot showing the absolute count of PLT-EVs in SARS-CoV-2+ patients enrolled during the 3rd wave (*n* = 78) and HC (*n* = 27); black dots indicate patients hospitalized for one week; empty boxes show mean ± SD; (b) PLT-EV counts at T0, stratified accordingly to prehospital treatments with/without heparin and steroids. For statistical analysis, D'Agostino and Pearson normality test was used before to perform Mann–Whitney and Kruskal-Wallis test. ^∗∗∗^*p* < 0.001.

We found that PLT-EV counts were higher in SARS-CoV-2 patients compared to HC, regardless of heparin or steroid prehospitalization treatments (Figure 1). These results strongly confirmed our previous findings showing PLT-EVs as biomarker of SARS-CoV-2 infection [8].

To investigate variation over time in PTL-EV counts, we monitored the kinetic of their absolute counts at the time of admission to the emergency room (T0) and one week after hospitalization (T7). 38/78 patients dropped out during the follow-up because of hospital discharge or death, and only 40/78 (black dots shown in Figure 1(a)) completed the study. 67.5% of the patients within this group were males (mean age 63.5 years (SD 14.1 years)), and the 4C mortality score [10], evaluated at T0, was 9.4 (SD 4.4); the median duration of symptoms before hospital admission corresponded to 7 days (Q1-Q3 4-9.5); percentage of SARS-CoV-2+ patients treated with steroids and heparin before hospital admission was 60% and 27.5%, respectively; 45% of patients were admitted to intensive care unit while 55% were admitted to other wards with intermediate intensity of care; the 30-day mortality was 17.5%.

**Figure 2:**
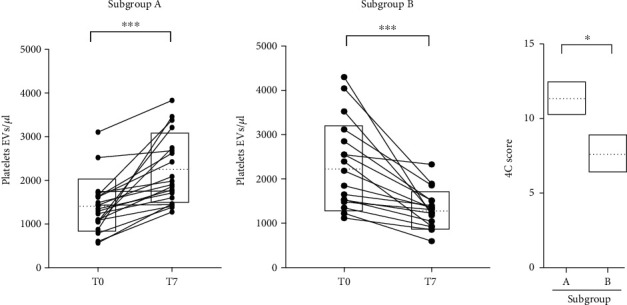
PLT-EV count is higher in more compromised SARS-CoV-2+ patients within one week of hospitalization. Line graphs identifying the two subgroups of patients (A and B, previously shown in Figure 1, black dots) showing either an increase or a decrease of PLT-EV counts at T7 and the 4C score evaluated at T0. For statistical analyses, D'Agostino and Pearson normality test was used before performing Wilcoxon sum rank and Mann–Whitney tests. ^∗∗∗^*p* < 0.001 and ^∗^*p* < 0.05.

With regard to the absolute count of PLT-EVs measured over time, we identified two different patterns in our population: 23 out of 40 patients (subgroup A) showed a statistically significant increase of PLT-EVs from T0 to T7, while 17 out of 40 patients (subgroup B) showed a significant decrease (Figure 2).

Interestingly, by stratifying patients based on their 4C mortality score [4] calculated at T0, we found that more compromised patients presented a significant increase of PLT-EV count during the hospital stay (subgroup A) (Figure 2).

## 4. Discussion

PLT-EVs, the most abundant circulating EVs, have been found to be increased in several human diseases, including viral infections, such as influenza and HIV [11–13]. In the former, it has been suggested that influenza H1N virus would activate PLTs which in turn release EVs [11]; in the latter, PLT-EVs might act as shuttle in the propagation of the virus [13]. PLT-EVs have been shown to contain SARS-CoV-2 RNA [14], as well as the exosomal cargo, and it was suggested that the virus might use the endocytosis route to spread infection [15].

Recently, we showed that the PLT-EV count is higher in SARS-CoV-2+ patients admitted to the emergency room, in comparison with SARS-CoV-2- patients and HC. We demonstrated that PLT-EVs also have a good performance as a diagnostic biomarker in discriminating SARS-CoV-2+ from SARS-CoV-2 patients, and we hypothesized that they might be involved in thromboembolism and vascular leakage, which are clinical hallmarks of SARS-CoV-2 infection [8].

The involvement of PLT-EVs in COVID-19 has been also shown by four independent groups [14, 16–18]. Zaid et al. evaluated PLT-EVs in PLT-free plasma of SARS-CoV-2+ patients. They found that PLT-EV levels were increased in the nonsevere SARS-CoV-2+ group in comparison with severe one; however, upon normalization on PLT number, they found that PLT-EVs were also increased in severe patients [14]. Guervilly et al. showed that tissue factor bearing PLTs and EVs were higher in COVID-19 patients who require mechanical ventilation [17]. Another study showed that half of EV population in COVID-19 patients was of PLT origin, and their counts were increased in comparison with HC [18]. PLT-EV counts were also found to be even more increased 30-day postdischarge, after COVID-19 remission [16].

In this study, we confirmed our previous findings [8] and evaluated for the first time, the kinetic of PLT-EVs within the acute phase, during the hospital stay.

As a detection/quantification method for EVs, we applied flow cytometry combined to patented probes, which is a technique, among others, recommended by the International Society for Extracellular Vesicles (ISEV) [7]. We used a protocol already validated by other groups [19–22] and, recently, by us in COVID-19 [8]. This protocol shows the advantage to probe directly and quickly PLT-EVs in fresh unmanipulated blood, since platelets—especially when under stress conditions (i.e., caused by ultracentrifugation)—may become activated, thus releasing EVs *per se*. Also, PLT-EVs might be partially lost when plasma is double centrifuged to remove platelets or due to repeated freeze/thaw cycles on stored plasma specimens [23].

Considering that those stressful conditions could interfere with the biological significance of the results obtained, the use of unmanipulated blood would be preferable and may provide more reliable data, as we did in our work.

We identified two subgroups of patients showing either an increase or a decrease of PLT-EV counts after one week of hospitalization compared to PLT-EV counts assessed at the time of admission to the emergency room. Since the recent guidelines for COVID-19 suggested to consider the 4C mortality score [10], which was not evaluated by Zaid et al. or by us [14] previously, we applied it to this study. This score refers to patient demographics, clinical observations, and blood parameters that are commonly available at the time of hospital admission by accurately characterizing the population of patients at high risk of death in hospital [10]. Interestingly, we found that more compromised patients (with an increased 4C mortality score) presented a significant increase of PLT-EV counts during the hospital stay.

During the COVID-19 pandemic, the use of the anticoagulant heparin and the anti-inflammatory dexamethasone has been shown as promising tools for the management of severe COVID-19 patients' symptoms [24]. We documented that prehospital treatment with steroids or heparin did not interfere with PLT-EV counts. Since we did not find in our cohort any correlation between absolute PLT-EV and platelet counts or d-dimer values (not shown), our findings lead us to hypothesize that the increase in PLT-EVs was related to the inflammatory response triggered by SARS-CoV-2 infection, rather than to PLTs or coagulation activation, though the role of PLTs in thromboinflammation is well documented [25].

Antiphospholipid antibodies (aPL) are detected in nearly half of SARS-CoV-2+ patients, and their prevalence was shown to be even higher in severe ones; but it is still debated if aPL positivity is just an epiphenomenon of an upregulated inflammatory state triggered by COVID-19 or a true player in the thrombotic storm of severe forms [26]. Potentially, aPL may target PLT-EVs and thus contribute to inflammatory state in COVID-19.

A recent study reported a higher expression of platelet CD142 marker (i.e., tissue factor) onto surface of serum-derived EVs in SARS-CoV-2+ patients compared with SARS-CoV-2-, both developed pneumonia. Interestingly, CD142 displayed higher biological activity only in SARS-CoV-2+ patients [27]. These findings might suggest that PLT-EVs initiate the extrinsic pathway of coagulation and, thereby, directly contribute to the high thrombotic risk in COVID-19. Lastly, since PLT-EVs are negatively charged, they may sustain the propagation of coagulation [28].

## 5. Conclusions

Our findings suggest a relationship between prognosis of SARS-CoV-2 and absolute count of PLT-EV absolute count and kinetics: these may be seen as biomarkers to monitor severity of SARS-CoV-2 and/or pathogenetic actors. By applying our fast and reliable method for EV count, which could easily be implemented in hospital clinical laboratories, PTL-EV counts could be translated into clinical practice. Of note, our method is not intended to replace PCR for viral quantification but it can rather represent a tool for clinicians to predict worsening of COVID-19 condition. Nevertheless, we firmly believe that further studies, in different and bigger cohorts, are still needed to confirm our data and clarify the potential pathophysiological role of PLT-EVs in the development of COVID-19.

## Figures and Tables

**Table 1:** Demographic and clinical features of hospitalized SARS-CoV-2+ patients.

	Median (Q1-Q3)
Age (years)	67 (58-80)
Gender (M/F), *N* (%)	55/23 (71%)
WBC^1^ (×10^3^/*μ*L)	8.61 (6.36-11.26)
Lymphocytes (×10^3^/*μ*L)	0.81 (0.59-1.13)
Creatinine (mg/dL)	0.86 (0.74-1.16)
AST^2^ (mU/mL)	39 (30-49)
ALT^3^ (mU/mL)	34.5 (20-47.5)
CRP^4^ (mg/dL)	6.44 (2.48-13.95)
PCT^5^ (ng/mL)	0.13 (0.05-0.28)
PLTs (×10^3^/*μ*L)	216 (173-300)
PT-INR^6^	1.02 (0.96-1.08)
Ferritin (ng/mL)	564 (171-1106)
LDH^7^ (U/L)	587.5 (495-752)
D-dimer (*μ*g/L)	1070 (609-1471)
IL^8^-6	14.75 (6.4-27)
SpO_2_/FiO_2_	427.5 (400-447)
PaO_2_/FiO_2_	247 (214-295)
SpO_2_	89 (85-94)
FiO_2_	21 (21-21)
Respiratory rate	25 (15-30)

^1^White blood cell, ^2^aspartate aminotransferase, ^3^alanine aminotransferase, ^4^C-reactive protein, ^5^procalcitonin, ^6^prothrombin time-international normalized ratio, ^7^lactate dehydrogenase, and ^8^interleukin.

## Data Availability

The data that support the findings of this study are available upon request from the corresponding author.
